# The Effect of Sodium Benzoate on the Gut Microbiome Across Age Groups

**DOI:** 10.3390/foods14172949

**Published:** 2025-08-24

**Authors:** Johanna M. S. Lemons, Jenni Firrman, Karley K. Mahalak, LinShu Liu, Adrienne B. Narrowe, Stephanie Higgins, Ahmed M. Moustafa, Aurélien Baudot, Stef Deyaert, Pieter Van den Abbeele

**Affiliations:** 1 United States Department of Agriculture, Agricultural Research Service, Eastern Regional Research Center, Dairy and Functional Foods Research Unit, 600 East Mermaid Lane, Wyndmoor, PA 19038, USA; 2 Division of Gastroenterology, Hepatology, and Nutrition, The Children’s Hospital of Philadelphia, Philadelphia, PA 19104, USA; 3 Department of Pediatrics, Perelman School of Medicine, University of Pennsylvania, Philadelphia, PA 19104, USA; 4 Center for Microbial Medicine, Children’s Hospital of Philadelphia, Philadelphia, PA 19104, USA; 5 Cryptobiotix, Technologiepark-Zwijnaarde 82, 9052 Ghent, Belgium

**Keywords:** sodium benzoate, benzoate, food additives, food preservatives, antimicrobial agent, gut microbiome

## Abstract

The food additive sodium benzoate (SB) has been used for decades as an antimicrobial to prevent food spoilage. SB has been deemed to pose no risk to human health when consumed at levels under 5 mg/kg body weight per day; however, when many of the supporting studies were conducted, the importance of the gut microbiome to human health was not yet appreciated. Given SB’s known antimicrobial qualities, it is important to assess the effect of this food additive on the human gut microbiome. The ex vivo SIFR^®^ (Systemic Intestinal Fermentation Research) technology was used to test the effect of SB on microbial communities from 24 donors, aged infants to older adults. A dose of 3.5 g/L SB elicited a drop in the Pseudomonadota phylum for multiple age groups but did not alter the alpha or beta diversity within any of these groups. This was accompanied by changes in the functional outputs that included an overall rise in butyrate and a drop in propionate production. This higher butyrate correlates with an increase in the abundance of several known butyrate producers in the presence of SB, although the genetic potential for its production in the community did not change. Overall, despite using a dose ten times higher than the accepted daily intake limit, the effect on the gut microbiome was minimal.

## 1. Introduction

Additives are allowed in foods under tightly regulated amounts based on extensive safety testing [1]. However, a safety aspect that has been historically absent from testing is the effect these food additives have on the human gut microbiome. This is because it has only recently become evident how important the gut microbiome is to human health. Newer studies, however, have made it clear that some commonly used emulsifiers can have a significantly detrimental effect on the composition of the gut microbiota [2,3,4], yet the effect of most additives still needs to be tested.

One of the most widely used food preservatives is sodium benzoate (SB) which prevents spoilage through its antimicrobial properties. Undissociated benzoic acid is the active form of this preservative and is the naturally occurring form present in some berries and spices, but its use in the food industry is limited by its poor solubility [5]. SB on the other hand, is highly soluble, but the amount present as the active form is pH dependent limiting its use to naturally acidic foods and drinks since it is ineffective in solutions with a pH above 5 [6]. The World Health Organization (WHO) acceptable daily intake (ADI) for benzoic acid and SB is 0–5 mg/kg body weight. As an antimicrobial agent in foods, it is allowed at a maximum level of 0.1% by weight in the United States [7]. SB is generally recognized as safe by the United States Food and Drug Administration (FDA) though there are reports of allergic responses in some sensitive individuals [7,8].

After ingestion, SB dissociates into its constituent ions in the small intestine and is rapidly absorbed in the proximal gastrointestinal tract. In animals and humans, it is believed that close to 99% of consumed benzoates are metabolized by the liver and excreted in urine as glycine conjugates, mainly hippuric acid (*N*-Benzoylglycine) [8]. Given this, it is unlikely that large amounts of SB will ever reach the colon and interact with the large microbial population residing there. Animal studies indicate that supplementation of feed with SB in place of antibiotics can improve intestinal health and growth rates of piglets, an effect that is thought to be mediated by its impact on the gut microbiota [9]. It is, therefore, still unclear how much interaction there is between benzoates and the gut microbiota in vivo.

Given its routine consumption, its known antimicrobial activity, and its potential to enter the colon, albeit small, it is important to assess the effect of SB on the gut microbial community. The microbiomes of infants and toddlers are still developing so factors that perturb natural development may have lifelong consequences. Indeed, epidemiological studies have established that individuals who experience alterations in their bacterial communities as children are at increased risk of some diseases later in life [10]. It is therefore very important to assess the effect of SB on both developing and stable microbiomes. In addition, it has been predicted that children may ingest more SB than adults due to a higher consumption of flavored drinks so it is especially important to assess the risk in this younger population [8]. Many current in vitro models used to study human gut microbiota are time consuming and low throughput making it difficult to quickly obtain representative insights. Additionally, some in vitro models fail to accurately recapitulate the in vivo microbiota composition [11,12,13]. On the other hand, the ex vivo SIFR^®^ (Systemic Intestinal Fermentation Research) technology is a high-throughput gut model with a demonstrated ability to accurately preserve in vivo-derived microbiota and provide predictive insights at the species level within 24–48 h [14].

In this study, the bioreactor-based SIFR^®^ technology in prism mode was applied to assess the impact of an SB dose ten times higher than the maximum ADI on the structure and function of the gut microbiota of 24 donors belonging to four age groups. This dose was chosen to search for effects above the ADI to have a built-in safety factor while also maximizing the potential to elicit a detectable effect with a dose that is still physiologically possible. The age groups were breastfed (BF) infants, toddlers, adults (25–40), and adults (60+). While the infants used in this study were breastfed, and while benzoate is a natural constituent of milk, it is unknown how maternal consumption of SB affects the levels in breastmilk [7]. Older BF infants also consume solid foods, which are a potential source of exposure to food additives like SB, making it difficult to assess a normal exposure level for this age group. Concerns over this were eliminated by using the same dose for all age groups. These groups were selected because they represent the transition from an infant community into adulthood. The purpose of this work was to (1) identify SB-directed changes to the gut microbiome and (2) distinguish whether age was a factor. This was accomplished using shotgun metagenomics and targeted metabolomics to identify the effects of high-dose SB across these four age groups.

## 2. Materials and Methods

### 2.1. Ex Vivo Culturing Experiments

Fecal samples from 6 donors (2 males and 4 females) within the following four specific age groups were harvested (24 donors total): BF infants (0–6 months), toddlers (2–4 years), adults (25–40 years), and adults (60+ years). Collection followed an IRB protocol approved by the Ethics Committee of the University Hospital Ghent, Belgium (BC-09977). The following criteria were used to select for donors: non-smokers, no GIT disorders or cancer, taking no medicine for allergies or psychological disorders, drinking less than three servings of alcohol/day, having consumed no anti-/pre-/probiotics for at least three months before their donations, and having a BMI of 20–25 (adults) [15].

The ex vivo SIFR^®^ technology was applied for gut microbiota testing as described previously [14]. Briefly, bioreactors containing anaerobically prepared nutritional media (Cryptobiotix, Ghent, Belgium) were inoculated with fecal slurry. For each donor, two bioreactors containing nutritional media were inoculated; one contained nutritional media only (no substrate control—NSC), and one was supplemented with 3.5 g/L SB (experimental). The dose of 3.5 g/L SB was selected to simulate a human dose that is ten times higher than the ADI for a 70 kg individual in order to capture effects outside the legal limits [16]. Environmental pH and gas production during the experiment were measured. At 0 and 24 h post-inoculation, samples were harvested for flow cytometry, metabolite analysis, and shotgun sequencing. All chemicals and reagents were purchased from Sigma Aldrich (St. Louis, MO, USA).

### 2.2. Bacterial Cell Counts

Total bacteria were enumerated using flow cytometry, as previously reported [17]. Briefly, samples were first diluted in anaerobic phosphate-buffered saline and then stained with 1 μM SYTO 16. A BD FACS Verse flow cytometer (BD, Erembodegem, Belgium) was used for counting and data were analyzed using FlowJo, version 10.8.1 [17].

### 2.3. Environmental pH, Gas Measurement, and Short-Chain Fatty Acid (SCFA) and Branched-Chain Fatty Acid (BCFA) Quantification

At the beginning and end of the experiment, a pH electrode was used to determine the environmental pH for each anaerobic culture tube and pressure in the headspace of each vessel was used to determine gas production. Diethyl ether was used to extract SCFAs and BCFAs which were quantified with GC flame ionization detection (Trace 1300, Thermo Fisher Scientific, Merelbeke, Belgium) as described previously [18]. The targeted method quantified the following specific SCFAs: acetate, propionate, butyrate, and valerate, as well as the following BCFAs: isobutyrate, isovalerate, and isocaproate and lactate. Total SCFA and BCFA amounts were calculated by summing the respective fatty acids.

### 2.4. DNA Extraction, Library Preparation, and Sequencing

DNA extraction, library preparation, and sequencing were performed at the Penn CHOP Microbiome Center according to a standard protocol as follows: DNA was extracted using the MagBeads FastDNATM Kit for Soil (MPBio), Irvine, CA, USA), quantified using the Quant-iT PicoGreen dsDNA assay kit (Thermo Fisher Scientific), followed by shotgun library preparation from 7.5 ng DNA using Illumina DNA Prep Library Prep kit (San Diego, CA, USA) and IDT for Illumina unique dual indexes at 1:4 scale reaction volume. Library preparation was assessed for quality using a Quant-iT PicoGreen dsDNA assay and samples that had library yields < 1 ng/μL were re-prepped. Libraries were pooled using equal volumes of each sample and sequenced using a 300-cycle Nano kit on the Illumina MiSeq (San Diego, CA, USA) to generate demultiplexing statistics to be used in re-pooling for the final sequencing library. Final library QC was performed to check size distribution and absence of additional adaptor fragments using an Agilent Bio Analyzer (Santa Clara, CA, USA). Finally, libraries were sequenced on an Illumina Novaseq 6000 v1.5 flow cell which produced 2 × 150 bp paired-end reads. Contamination due to the environment and reagent contamination was empirically assessed by processing extraction blanks and nucleic acid-free water along with experimental samples. Positive sequencing controls included DNA from *Vibrio campbellii* and Lambda phage.

### 2.5. Read-Based Taxonomic and Functional Profiling

Preprocessing of raw shotgun metagenomic sequencing data to remove sequencing adapters, PhiX, other artifacts, and human host reads (GRCh37) was performed using BBDuk v. 39.01. [19]. BBDuk quality trimming used parameters (k = 31, hdist = 1, ftm = 5) [19]. Trimmed and filtered reads were input to MetaPhlAn v 4.1.1 (using the vJun23_CHOCOPhlAnSGB_202403 reference database) [20]. Flow cytometry counts were used to normalize abundances producing final taxonomic abundance profiles for use in downstream analysis.

HUMAnN (HMP Unified Metabolic Analysis Network) v.4.0.0a1 was used to perform read-based functional profiling [21]. Profiles were converted to EC (Enzyme Commission number), KEGG (Kyoto Encyclopedia of Genes and Genomes) ortholog, and PFAM (Protein Families database) identifiers and were then normalized to relative abundance (TSS (Total Sum Scaling) sum = 1) or CoPM (copies per million, TSS sum = 1 M). Differential pathway and function abundance testing was performed using MaAsLin3 (Microbiome Multivariable Associations with Linear Models) [22] using treatment and age as fixed effects and using donor as random effect. Results with false-discovery rate corrected *p*-value < 0.05 were retained for analysis.

The MetaPhlAn (Metagenomics Phylogenetic Analysis) utility script calculated diversity. R was used to calculate alpha diversity (taxonomic richness and Shannon’s index) and beta diversity (weighted and unweighted UniFrac metrics). Beta diversity matrices were plotted using principal coordinate analysis (PCoA). Significant differences among treatments and age groups for alpha diversity metrics were determined using paired Wilcoxon signed-rank test. Testing for significant clustering by either treatment or age for both the weighted and unweighted UniFrac distance matrices was performed using PERMANOVA (pairwise adonis2 package) [23].

### 2.6. Taxon and Pathway Association Testing

For the taxonomic data, log-transformed data (FC-corrected abundance data for taxon, and CPM normalized for functional data) was used for all 24 h samples using ‘donor’ as random effects and product as the fixed effect, specifying ‘NSC’ as the reference level according to the per-feature model: feature ~ (intercept) + SB + (1|donor), where feature is either taxon or pathway depending on the dataset. Multiple testing correction was performed by applying the Benjamini-Hochberg method with the method’s default FDR threshold of 0.25.

### 2.7. Other Analyses and Visualizations

Visualization and further statistical analyses were performed in R/RStudio (v.4.1.3) using the following packages: tidyverse (v.1.3.1) [24], vegan (v.2.6-2) [25], and ape (v.5.6-2) [26]. GraphPad Prism 10 was used to create bacterial abundance plots (GraphPad Software, San Diego, CA, USA).

## 3. Results

**Figure 1:**
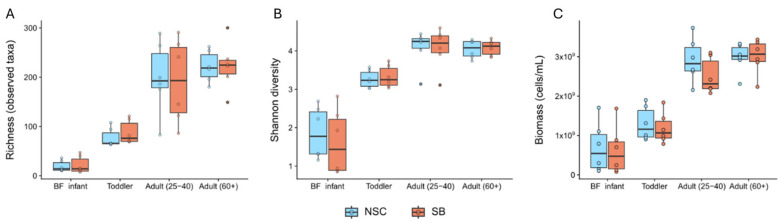
Intrasample diversity metrics, (**A**) species richness and (**B**) Shannon’s diversity, and (**C**) bacterial cell count data for the different age groups incubated with SB or under control conditions. None of the age groups exhibited a statistically significant change in response to treatment with SB as assessed by a two-sided paired Wilcoxon signed-rank test. When age was eliminated as a variable, there was a statistically significant drop in cell counts after SB treatment (*p* adj = 0.014).

The composition and functional potential of gut microbial communities harvested from 24 individuals in four age groups were analyzed using metagenomic sequencing and metabolite data. The effect of SB on intrasample (alpha) and intersample (beta) diversity metrics was compared against media-only controls (NSC). Alpha diversity was assessed using both taxon richness and the Shannon index. Figure 1 shows that, as expected, adults have higher species richness and community diversity than toddlers and infants, but SB has no effect on either of these metrics for any age group. This indicated that the taxonomic composition of the bacterial communities and bacterial abundances within the communities were largely unaffected by treatment with SB. While there was a trend toward decreased cell counts for BF infants, toddlers, and adults (25–40) in the presence of SB, this difference only becomes statistically significant when all donors are considered together. Separately, there were not enough replicates within each age group to assign significance to such small differences.

**Figure 2:**
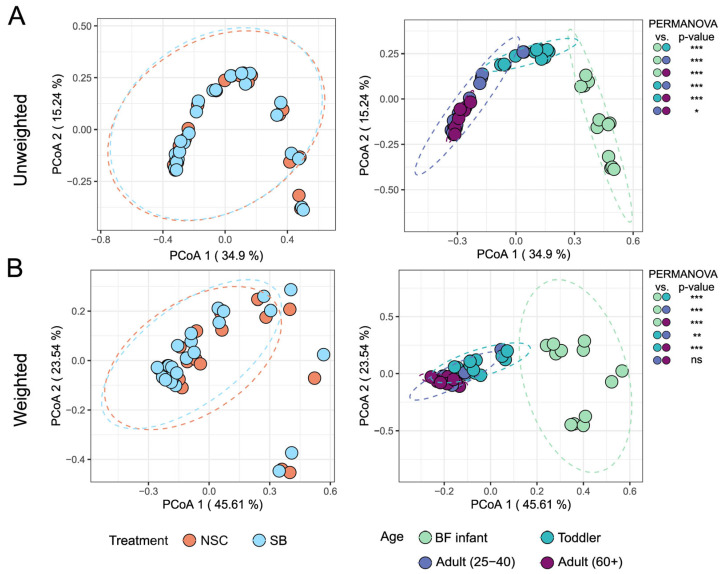
Intersample diversity represented in identical (**A**) unweighted and (**B**) weighted UniFrac distance plots, colored differently to emphasize either treatment effect (left panel), or age effect (right) (n = 6 NSC and n = 6 SB for each of the four age groups). The zoom varies slightly to accommodate the ellipses indicating the 95% confidence interval. PERMANOVA (pairwise adonis2 package) was used to test for significant clustering by either treatment or age for both the weighted and unweighted UniFrac distance matrices. (*** = *p*-value < 0.001, ** = *p*-value < 0.01, * = *p*-value < 0.05).

The beta diversity of the treated and untreated communities for each age group was evaluated using unweighted and weighted UniFrac distances and depicted using a Principal Coordinate Analysis (PCoA) plot, shown in Figure 2. Unweighted UniFrac distances consider only the presence or absence of taxa, while weighted ones also consider absolute abundance. The unweighted metric is more sensitive for uncovering differences derived from low-abundant taxa, while the weighted metric can reveal community differences that are due to changes in relative taxon abundance [27]. Their combined use on the same data set can allow for varying conclusions to be drawn about the nature of community differences. In this study, however, both metrics indicated that SB treatment did not affect the beta diversity of the communities for any age group, but the communities derived from different age groups were significantly different from each other. The exception is that the weighted distances failed to show a statistically significant difference between the two adult cohorts.

**Figure 3:**
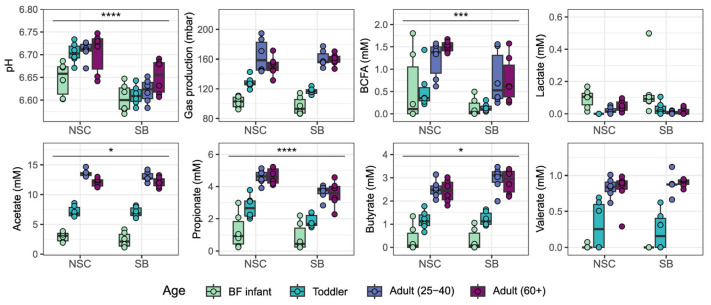
Functional readouts for the NSC- and SB-treated bacterial communities for each age group. Statistical significance was assessed using a two-sided paired Wilcoxon signed-rank test. SB-induced changes only reached significance for some of the parameters when all donors were considered together as indicated (**** = *p*-value < 0.0001, *** = *p*-value < 0.001, * = *p*-value < 0.05).

The fermentative capacity of the different communities was assessed by measuring the levels of various byproducts of this process. In general, fermentation of fiber leads to the production of gas and SCFAs whereas BCFAs often arise from protein fermentation [28,29]. As has been observed previously, the amounts of fermentation products did vary by age group [15]. The amounts of the four measured SCFAs, gas, and BCFAs were all increased with age. Conversely, lactate production was highest in infants. There was, however, no significant change in the level of any of these byproducts in response to SB treatment for any individual age group (Figure 3). As was the case with cell counts, the difference between SB and NSC was only significant for some byproducts when all donors were considered together. Gas, valerate, and lactate production were unaffected by SB, while acetate, propionate, and BCFAs decreased, and butyrate increased. Since there was no compelling evidence to suggest that age groups were differentially affected by SB, all further statistical analyses only considered the effect of SB across all age groups.

**Figure 4:**
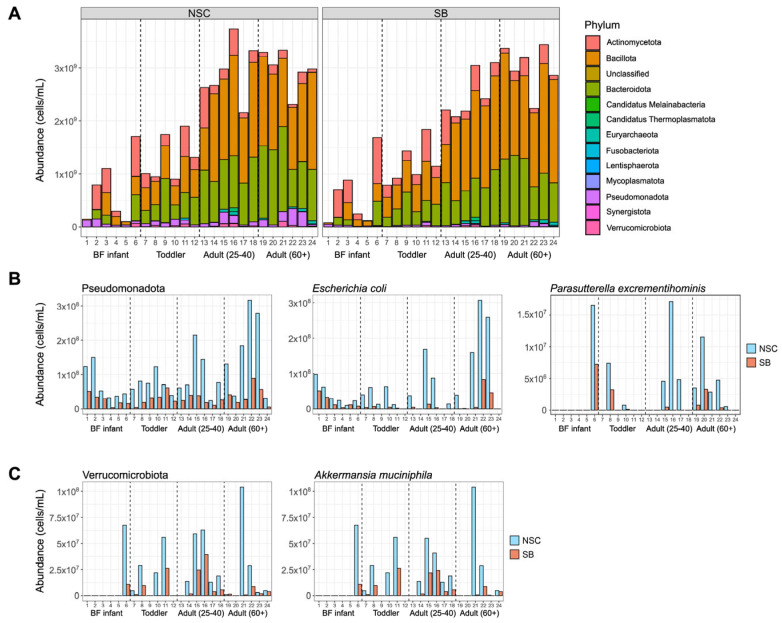
Taxonomic changes resulting from SB treatment including (**A**) absolute abundance of all phyla present in this study and (**B**,**C**) abundance plots for the species that contributed the most to the significant changes in Pseudomonadota and Verrucomicrobiota (q-value < 0.05, MaAsLin3).

Metagenomic sequencing data was used to look at the taxonomic distributions at the phylum, genus, and species levels to get an increasingly granular look at what happened to the communities treated with SB. Bacteria and archaea originating from 13 different phyla were identified in the microbial communities in this study and most belonged to the Actinomycetota, Bacillota, Bacteroidota, and Pseudomonadota (previously named Actinobacteria, Firmicutes, Bacteroidetes, and Proteobacteria, respectively) (Figure 4A). Only three archaeal species were detected and only in adult communities. Pseudomonadota and Verrucomicrobiota were the only phyla significantly (individual q-value < 0.05) decreased by SB treatment when age was not considered (Figure 4B,C). Only Pseudomonadota was significantly different for infants and adults (both age groups), but not toddlers, reinforcing the decision to focus on large effects seen across all age groups.

There were 16 genera significantly affected by SB treatment, most of which are represented by the starred species in Table 1, which shows all species that changed significantly (q-value < 0.05) with SB treatment. The four genera that are not represented in Table 1 are *GGB9747*, *Holdemania*, *Lachnospira*, and *Anaerotignum*. *GGB9747* and *Anaerotignum* each had one species which tended to account for most of the change, while *Holdemania* and *Lachnospira* were represented by multiple species that all exhibited smaller changes in the same direction. At the species level, there were two within Pseudomonadota that exhibited large, significant decreases with SB treatment: *Escherichia coli* and *Parasutterella excrementihominis* (Figure 4B and Table 1). In the donors where these species were present and declining, on average they account for 70% of the total loss in this phylum, 63% of which can be attributed solely to *E. coli*. Other Pseudomonadota, including *Citrobacter freundii*, *Klebsiella michiganensis*, and *Enterobacter hormaechei*, also exhibited large decreases in the presence of SB but these taxa were present in only a few donors and the changes did not reach statistical significance (Supplemental Data). Approximately 93% of the drop in Verrucomicrobiota can be attributed to the large drop in the abundance of *Akkermansia muciniphila* with SB treatment (−2.27 log2 fold change, q-value = 0.0985) (Figure 4C).

**Figure 5:**
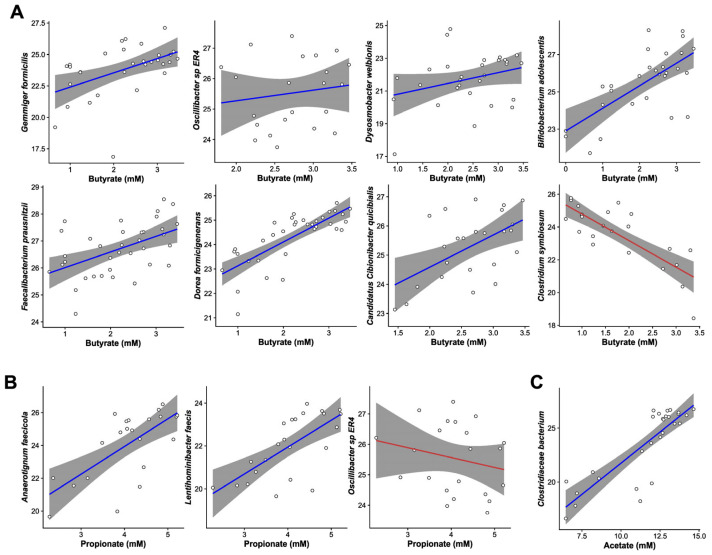
Generalized linear models generated by MaAsLin3 showing significant associations (q-value < 0.05) between species and (**A**) butyrate, (**B**) propionate, and (**C**) acetate. A blue line indicates a positive correlation, and a red line indicates a negative correlation.

MaAsLin3-generated generalized linear models were used to identify relationships between species-level abundance and SCFA concentrations. Figure 5A–C shows a selection of the species that were significantly associated with butyrate, propionate, and acetate (respectively) which had at least 10 observations per category (SB and NSC) and a coefficient > 0.5. The slope of the line, which was generated using GLM smoothing of the scatter plot data, indicates whether the correlation was positive or negative. There were ten species whose abundance significantly correlated with changes in butyrate (qval_individual < 0.05) including *Gemmiger formicilis*, *Oscillibacter* sp. *ER4*, *Dysosmobacter welbionis*, *Faecalibacterium prausnitzii*, and *Candidatus Cibionibacter quicibialis* from Table 1. All five species whose abundance significantly correlated with changes in propionate were also present in Table 1 (*A. faecicola*, *Lentihominibacter faecis*, *C. quicibialis*, *F. prausnitzii*, and *Oscillibacter* sp. *ER4*). Although there were four species whose abundance significantly correlated with changes in acetate, none of those species changed significantly.

Metagenomics sequencing data revealed that 2165 KEGG orthologs (KO) were significantly (q < 0.05) affected by SB (1852 down, 313 up). Of these, 1732 were changed ≥1 fold. Among these was K05782 (−2.56 log2 fold change, q-value = 2.95 × 10^−4^), a benzoate membrane transport protein which is known to be expressed by *E. coli*. The KO changes also correspond to 130 large changes to MetaCyc pathways that were significantly (adjusted *p*-value < 0.05) affected by SB (Supplemental Data). 114 of the changes in pathway abundance can be at least partially attributed to a loss of *E. coli.* Many of these pathways are also found in the genomes of *P. excrementihominis*, *C. freundii*, *K. michiganensis*, *E. hormaechei*, and *A. muciniphila* which were also depleted by SB treatment. Ten of the remaining pathways could not be attributed to any particular taxon and the other six are shown in Figure S1, some of which were encoded by taxa in Table 1, suggesting that changes in these taxa may have driven the changes observed in these pathways. The only named species harboring PWY-821 (super pathway of sulfur amino acid biosynthesis) and PWY-7269 (mitochondrial NADPH production) were *A. muciniphila* and *Phocaeicola dorei*, respectively, both of which were decreased with SB. PWY-6953 (dTDP-3-acetamido-&alpha;-D-fucose biosynthesis) was encoded by several taxa including *Bacteroides ovatus* which was significantly reduced with SB treatment. Changes in PWY-7942 (oxi-L-proline metabolism) and PWY-801 (homocysteine and cysteine interconversion) were at least partially explained by the loss of *C. freundii*, *K. michiganensis,* and *E. hormaechei.* Interestingly another species attributed with harboring PWY-801 was the archaea, *Candidatus Methanomethylophilus alvus*. This species was also the only named taxa containing the final pathways, PWY-6293 (super pathway of L-cysteine biosynthesis); however, it was only identified in one adult donor.

There were also smaller, not statistically significant changes to other KOs involved in MetaCyc pathways that could be attributed to taxa other than *E. coli*. Among those, the abundance of some SCFA production pathways in the communities was slightly altered including P108-PWY: pyruvate fermentation to propanoate I, PWY-5494: pyruvate fermentation to propanoate II (acrylate pathway), PWY-5677: succinate fermentation to butanoate, and PWY-5130: 2-oxobutanoate degradation I (Supplemental Data). PWY-5130 was encoded by over 40 species within the communities of this study including *Alistipes putredinis*, *Bacteroides ovatus*, *Bacteroides thetaiotaomicron*, *D. welbionis*, *Odoribacter splanchnicus*, and *P. dorei* from Table 1. P108-PWY was encoded by *A. muciniphila*, *B. ovatus*, *B. thetaiotaomicron*, *D. welbionis*, and others. Gene/pathway abundance is not a direct indication of enzymatic activity but many of the bacteria whose abundances correlate with SCFA levels (Figure 5) are already well-known SCFA producers so it is plausible that the alterations in SCFA levels can be explained by changes in the abundance of species encoding pathways that produce these byproducts.

## 4. Discussion

The antimicrobial activity of benzoate, like other weak acids used as food preservatives, is attributed to its ability to lower intracellular pH in organisms like *E. coli*, *Saccharomyces cerevisiae*, and *Zygosaccharomyces bailii*. In yeast, there is evidence that this leads to an inhibition of glycolysis, causing decreased ATP production and growth inhibition, whereas in *E. coli* glucose consumption remains high even when growth is inhibited [30,31,32]. While there is insufficient evidence from this study to support a specific mechanism, it does appear that *E. coli* is particularly susceptible to the bacteriostatic effects of SB. Among all the bacterial species that significantly changed in response to SB treatment, the effect on *E. coli* was the largest. This one species largely explains the observed drop in the Pseudomonadota phylum. In addition, most of the KOs and pathways that decrease were encoded by *E. coli*, among others, so it was not surprising that they went down when the number of this species was decreasing. The other phylum that was significantly reduced by SB was Verrucomicrobiota, and this could be attributed mostly to a loss of *A. muciniphila*. While some strains of *E. coli* are often considered as pathobionts and *A. muciniphila* is touted as a beneficial microorganism, they both are gram-negative bacteria that may share other commonalities that lead to increased susceptibility to SB [33]. Although the decrease in *A. muciniphila* did not achieve statistical significance, it could be considered a negative effect, since the abundance of this species is positively associated with host health [34].

While this study is the first to assess the effect of SB on bacterial communities from human donors, it is not the first to study its effect on gut microbes. A paper from 2018 explored the effect of SB and other food preservatives on 8 bacterial strains isolated from human feces grown axenically under vastly different growth conditions and concluded that exposure to the antimicrobial food additive SB may detrimentally affect the host [35]. Among their results was the observation that *Bifidobacterium longum* was more susceptible to the growth-inhibitory effects of SB than *E. coli*. A later study by this same group showed that combined treatment of the three most frequently used additives, sodium benzoate, sodium nitrite, and potassium sorbate, elicited dysbiosis in humanized Nod2-deficient mice [36]. While it is misleading to directly compare these studies to the SIFR^®^ study presented here, there is little agreement between the results. Using the SIFR^®^ technology to study the impact of SB revealed that *E. coli* levels dropped dramatically whereas *B. longum* levels slightly increased which directly opposes the in vitro study. Most likely, the discrepancy between the results is due to the differences between experimental conditions. Because the SIFR^®^ model cultivates a complete donor community under standardized, anaerobic conditions, it is a more robust model of the gut microbiota than the axenic cultures and is likely more indicative of what happens in vivo. A comparison with the humanized mice is confounded by the genetic background of the animals and the multiple additives tested at once, but the results are at odds with the results of the SIFR^®^ study. Dysbiosis is the term used to characterize a gut microbial signature that negatively correlates with human health and it is often identified as a decrease in species richness and, depending on disease, is associated with a rise in some taxa and a loss of others [37]. One of the taxonomic changes most often associated with dysbiosis is a rise in *E. coli* and other *Enterobacteriaceae*. Unlike the mouse study, the results of the SIFR^®^ study show that SB greatly reduces several members of this family.

There exists a large body of literature describing the effect of SB supplementation on farm animal growth and gut ecology that supports the conclusion that SB does not negatively impact the gut microbiota [9,38,39,40]. In young pigs and calves, SB supplementation increases the daily weight gain and feed conversion ratio while decreasing potential pathogens like *E. coli*. Some have attributed these beneficial effects to the action of SB on the small intestine microbiota which is logical given that the majority of SB is absorbed before entering the colon [41]. Regardless, results from these animal studies do agree with the results of the SIFR^®^ study in that they both demonstrate a particular susceptibility of coliform bacteria to the effects of SB. In addition, increased butyrate production was observed in the rumen of dairy calves given SB, just as butyrate levels in this study were higher after SB treatment [38]. SCFAs are known to be beneficial to host health [28,42]. Butyrate, in particular, is an important energy substrate for the colonic epithelium and serves an important role as a signaling molecule in the host. A change in the level of lactic acid bacteria has often been reported in studies from animals, though its directionality is unclear [9,43]. There were no significant changes in the canonical lactic acid bacteria in this study, but there were changes in several other species from the Bacillota phylum that both increased and decreased in response to SB.

Among the bacterial species that significantly changed in abundance after SB treatment were many whose levels also strongly correlated with the levels of one or more SCFAs. Using HUMAnN4, it was confirmed that some of these taxa do harbor the genetic machinery necessary to metabolize SCFAs. Many of these taxa are also already well-known butyrate producers including *Oscillibacter*, *D. welbionis*, and *F. prausnitzii* [44,45,46,47]. The species *C. quicibialis* is phylogenetically placed between *Faecalibacterium* and *Ruminococcus*, both of which contain butyrate producers, and may also produce butyrate [48]. The positive correlation seen between these species and butyrate suggested that their higher abundances may be causative of the increased butyrate seen after SB treatment even though the genetic potential for butyrate production was unchanged. Interestingly, when *G. formicilis* was first described, its ability to produce butyrate and formate without significant gas formation also agreed with other fermentation parameters from this study [44]. Gas formation was not increased in response to SB, but pH did decrease. Formate is not a metabolite routinely measured in this protocol so it is unknown whether the drop in pH can be partially attributed to this metabolite along with butyrate.

At a dose of 3.5 g/L, SB caused statistically significant reductions in the number of Pseudomonadota and Verrucomicrobiota without affecting the overall alpha and beta diversity of the communities. Of the over 900 species identified in this study, the abundance of only 16 of them was significantly altered by treatment with SB, though most of the changes in functional potential can be attributed to even fewer. While the observed changes in functional outputs did correlate with changes in the abundance of some of these species, they cannot be easily explained by changes in the genetic potential of the community.

## 5. Conclusions

In summary, the data from this study revealed that even when human colon communities from varied age groups were treated with a supraphysiologic dose of SB, the effect was very minimal. Some of the changes may even be beneficial, including a reduction in the levels of *Enterobacteriaceae* and an increase in butyrate. Combined with the fact that only a small portion of SB is thought to make it to the colon, it is unlikely that its consumption within the ADI will negatively impact the colonic ecosystem. Since it is suspected that SB influences animal health largely through its effect on the small intestine, future research may want to use an in vitro human small intestine model to study this further [49] or, as others have done, test the effect of SB in combination with other food additives commonly present in the diet.

## Figures and Tables

**Table 1:** Fold change and q-values for all 16 species whose abundances changed significantly (q-value < 0.05) in response to SB treatment along with the phylum data for those species. * Indicates species that represent genera that were also significantly changed by SB.

Phylum	Species	Log2fold Change	q-Value
Bacillota	*Ruminococcus lactaris*	1.71 ± 0.33	8.67 × 10^−3^
Bacillota	*Anaerostipes hadrus* *	1.42 ± 0.37	0.036
Bacillota	*Fusicatenibacter saccharivorans* *	0.96 ± 0.26	0.042
Bacillota	*Oscillibacter* sp. *ER4*	0.79 ± 0.15	0.013
Bacillota	*Gemmiger formicilis* *	0.72 ± 0.18	0.036
Bacillota	*Dysosmobacter welbionis* *	0.64 ± 0.15	0.021
Bacillota	*Faecalibacterium Prausnitzii* *	0.48 ± 0.07	8.26 × 10^−5^
Bacillota	*Candidatus Cibionibacter quicibialis* *	0.32 ± 0.04	1.33 × 10^−4^
Bacteroidota	*GGB1567 SGB2154* *	−0.26 ± 0.01	0.042
Bacteroidota	*Bacteroides ovatus*	−0.46 ± 0.12	0.042
Bacteroidota	*Alistipes putredinis* *	−0.53 ± 0.11	0.015
Bacteroidota	*Alistipes finegoldii* *	−0.85 ± 0.13	2.74 × 10^−3^
Bacteroidota	*Phocaeicola dorei* *	−1.17 ± 0.16	1.33 × 10^−4^
Bacillota	*Lentihominibacter faecis* *	−2.17 ± 0.41	9.13 × 10^−4^
Pseudomonadota	*Parasutterella excrementihominis* *	−2.26 ± 0.36	0.016
Pseudomonadota	*Escherichia coli* *	−2.89 ± 0.46	4.13 × 10^−4^

## Data Availability

Raw metagenomic sequencing data are available in the NCBI Sequence Read Archive associated with BioProject PRJNA1160256.

## Supplementary Materials

The following supporting information can be downloaded at: https://www.mdpi.com/article/10.3390/foods14172949/s1, Figure S1: Species-level relative contribution of taxa that encode the 6 pathways significantly changed by SB that can be attributed to specific taxa, but not *E. coli*; Table S1: Excel file containing species-level abundance; Table S2: Excel file containing identified KOs; TableS3: Excel file containing pathway data for SB-treated communities compared to NSC.

## Abbreviations

The following abbreviations are used in this manuscript:

SB	sodium benzoate
NSC	no substrate control
ADI	acceptable daily intake
BF	breastfed
SCFA	short-chain fatty acids
BCFA	branched-chain fatty acids
SIFR	Systemic Intestinal Fermentation Research
KO	KEGG ortholog
PCoA	principal coordinate analysis

## References

[1] EFSA Panel on Food Additives and Nutrient Sources added to Food (ANS) (2012). Guidance for submission for food additive evaluations. EFSA J..

[2] Chassaing B., Compher C., Bonhomme B., Liu Q., Tian Y., Walters W., Nessel L., Delaroque C., Hao F., Gershuni V. (2022). Randomized Controlled-Feeding Study of Dietary Emulsifier Carboxymethylcellulose Reveals Detrimental Impacts on the Gut Microbiota and Metabolome. Gastroenterology.

[3] Chassaing B., Koren O., Goodrich J.K., Poole A.C., Srinivasan S., Ley R.E., Gewirtz A.T. (2015). Dietary emulsifiers impact the mouse gut microbiota promoting colitis and metabolic syndrome. Nature.

[4] Panyod S., Wu W.K., Chang C.T., Wada N., Ho H.C., Lo Y.L., Tsai S.P., Chen R.A., Huang H.S., Liu P.Y. (2024). Common dietary emulsifiers promote metabolic disorders and intestinal microbiota dysbiosis in mice. Commun. Biol..

[5] Wibbertmann A., Kielhorn J., Koennecker G., Mangelsdorf I., Melber C., United Nations Environment Programme, and the World Health Organization (2000). Benzoic Acid and Sodium Benzoate.

[6] De Villiers M.M., Thompson J.E. (2009). Antimicrobial Preservatives. A Practical Guide to Contemporary Pharmacy Practice.

[7] International Program on Chemical Safety (1998). Concise International Chemical Assessment Document.

[8] EFSA Panel on Food Additives and Nutrient Sources (ANS) (2016). Scientific Opinion on the re-evaluation of benzoic acid (E 210), sodium benzoate (E 211), potassium benzoate (E 212) and calcium benzoate (E 213) as food additives. EFSA J..

[9] Guggenbuhl P., Séon A., Quintana A.P., Nunes C.S. (2007). Effects of dietary supplementation with benzoic acid (VevoVitall^®^) on the zootechnical performance, the gastrointestinal microflora and the ileal digestibility of the young pig. Livest. Sci..

[10] Tamburini S., Shen N., Wu H.C., Clemente J.C. (2016). The microbiome in early life: Implications for health outcomes. Nat. Med..

[11] O’Donnell M.M., Rea M.C., Shanahan F., Ross R.P. (2018). The Use of a Mini-Bioreactor Fermentation System as a Reproducible, High-Throughput ex vivo Batch Model of the Distal Colon. Front. Microbiol..

[12] Rajilić-Stojanović M., Maathuis A., Heilig H.G.H.J., Venema K., de Vos W.M., Smidt H. (2010). Evaluating the microbial diversity of an in vitro model of the human large intestine by phylogenetic microarray analysis. Microbiology.

[13] Van den Abbeele P., Grootaert C., Marzorati M., Possemiers S., Verstraete W., Gérard P., Rabot S., Bruneau A., El Aidy S., Derrien M. (2010). Microbial Community Development in a Dynamic Gut Model Is Reproducible, Colon Region Specific, and Selective forBacteroidetesandClostridiumCluster IX. Appl. Environ. Microbiol..

[14] Van den Abbeele P., Deyaert S., Thabuis C., Perreau C., Bajic D., Wintergerst E., Joossens M., Firrman J., Walsh D., Baudot A. (2023). Bridging preclinical and clinical gut microbiota research using the ex vivo SIFR^®^ technology. Front. Microbiol..

[15] Lemons J.M.S., Narrowe A.B., Firrman J., Mahalak K.K., Liu L., Higgins S., Moustafa A.M., Baudot A., Deyaert S., Van den Abbeele P. (2025). The food additive butylated hydroxyanisole minimally affects the human gut microbiome ex vivo. Food Chem..

[16] Joint FAO/WHO Expert Committee on Food Additives, WHO, Food and Agriculture Organization of the United Nations, International Programme on Chemical Safety Toxicological evaluation of certain food additives and contaminants. Proceedings of the 37th Meeting of the Joint FAO/WHO Expert Committee on Food Additives (JEFCA).

[17] Van den Abbeele P., Detzel C., Rose A., Deyaert S., Baudot A., Warner C. (2023). Serum-Derived Bovine Immunoglobulin Stimulates SCFA Production by Specific Microbes in the Ex Vivo SIFR^®^ Technology. Microorganisms.

[18] Van den Abbeele P., Deyaert S., Albers R., Baudot A., Mercenier A. (2023). Carrot RG-I Reduces Interindividual Differences between 24 Adults through Consistent Effects on Gut Microbiota Composition and Function Ex Vivo. Nutrients.

[19] Bushnell B. BBMap-Bushnell B.—Sourceforge.Net/Projects/Bbmap/. https://sourceforge.net/projects/bbmap/files/.

[20] Blanco-Miguez A., Beghini F., Cumbo F., McIver L.J., Thompson K.N., Zolfo M., Manghi P., Dubois L., Huang K.D., Thomas A.M. (2023). Extending and improving metagenomic taxonomic profiling with uncharacterized species using MetaPhlAn 4. Nat. Biotechnol..

[21] Beghini F., McIver L.J., Blanco-Miguez A., Dubois L., Asnicar F., Maharjan S., Mailyan A., Manghi P., Scholz M., Thomas A.M. (2021). Integrating taxonomic, functional, and strain-level profiling of diverse microbial communities with bioBakery 3. eLife.

[22] Nickols W.A., Kuntz T., Shen J., Maharjan S., Mallick H., Franzosa E.A., Thompson K.N., Nearing J.T., Huttenhower C. (2024). MaAsLin 3: Refining and extending generalized multivariate linear models for meta-omic association discovery. bioRxiv.

[23] Martinez Arbizu P. (2020). Pairwiseadonis: Pairwise Multilevel Comparison Using Adonis. R Package Version 0.4. https://github.com/pmartinezarbizu/pairwiseAdonis.

[24] Wickham H. (2009). Ggplot2: Elegant Graphics for Data Analysis.

[25] Oksanen J.S.G., Blanchet F., Kindt R., Legendre P., Minchin P., O’Hara R., Solymos P., Stevens M., Szoecs E., Wagner H. (2022). Weedon Vegan: Community Ecology Package.

[26] Paradis E., Schliep K. (2019). ape 5.0: An environment for modern phylogenetics and evolutionary analyses in R. Bioinformatics.

[27] Lozupone C.A., Hamady M., Kelley S.T., Knight R. (2007). Quantitative and qualitative beta diversity measures lead to different insights into factors that structure microbial communities. Appl. Env. Microbiol..

[28] Koh A., De Vadder F., Kovatcheva-Datchary P., Bäckhed F. (2016). From Dietary Fiber to Host Physiology: Short-Chain Fatty Acids as Key Bacterial Metabolites. Cell.

[29] Russell W.R., Gratz S.W., Duncan S.H., Holtrop G., Ince J., Scobbie L., Duncan G., Johnstone A.M., Lobley G.E., Wallace R.J. (2011). High-protein, reduced-carbohydrate weight-loss diets promote metabolite profiles likely to be detrimental to colonic health. Am. J. Clin. Nutr..

[30] Salmond C.V., Kroll R.G., Booth I.R. (1984). The Effect of Food Preservatives on pH Homeostasis in *Escherichia coli*. Microbiology.

[31] Krebs H.A., Wiggins D., Stubbs M., Sols A., Bedoya F. (1983). Studies on the mechanism of the antifungal action of benzoate. Biochem. J..

[32] Warth A.D. (1991). Mechanism of action of benzoic acid on Zygosaccharomyces bailii: Effects on glycolytic metabolite levels, energy production, and intracellular pH. Appl. Environ. Microbiol..

[33] Kolmos H.J. (1976). The bactericidal action of benzoic acid and sodium acetate on the gram-negative flora of dialysis fluid. Acta Pathol. Microbiol. Scand B.

[34] Ioannou A., Berkhout M.D., Geerlings S.Y., Belzer C. (2025). Akkermansia muciniphila: Biology, microbial ecology, host interactions and therapeutic potential. Nat. Rev. Microbiol..

[35] Hrncirova L., Hudcovic T., Sukova E., Machova V., Trckova E., Krejsek J., Hrncir T. (2019). Human gut microbes are susceptible to antimicrobial food additives in vitro. Folia Microbiol..

[36] Hrncirova L., Machova V., Trckova E., Krejsek J., Hrncir T. (2019). Food Preservatives Induce Proteobacteria Dysbiosis in Human-Microbiota Associated Nod2-Deficient Mice. Microorganisms.

[37] Wei S., Bahl M.I., Baunwall S.M.D., Hvas C.L., Licht T.R., Drake H.L. (2021). Determining Gut Microbial Dysbiosis: A Review of Applied Indexes for Assessment of Intestinal Microbiota Imbalances. Appl. Environ. Microbiol..

[38] Dai H., Huang Q., Li S., Du D., Yu W., Guo J., Zhao Z., Yu X., Ma F., Sun P. (2024). Effect of Dietary Benzoic Acid Supplementation on Growth Performance, Rumen Fermentation, and Rumen Microbiota in Weaned Holstein Dairy Calves. Animals.

[39] Giannenas I., Papaneophytou C.P., Tsalie E., Pappas I., Triantafillou E., Tontis D., Kontopidis G.A. (2014). Dietary Supplementation of Benzoic Acid and Essential Oil Compounds Affects Buffering Capacity of the Feeds, Performance of Turkey Poults and Their Antioxidant Status, pH in the Digestive Tract, Intestinal Microbiota and Morphology. Asian-Australas. J. Anim. Sci..

[40] Gong H., Yang Z., Celi P., Yan L., Ding X., Bai S., Zeng Q., Xu S., Su Z., Zhuo Y. (2021). Effect of benzoic acid on production performance, egg quality, intestinal morphology, and cecal microbial community of laying hens. Poult. Sci..

[41] Choi H., Kim S.W. (2024). Dietary Intervention of Benzoic Acid for Intestinal Health and Growth of Nursery Pigs. Animals.

[42] Morrison D.J., Preston T. (2016). Formation of short chain fatty acids by the gut microbiota and their impact on human metabolism. Gut Microbes.

[43] Chen J.L., Zheng P., Zhang C., Yu B., He J., Yu J., Luo J.Q., Mao X.B., Huang Z.Q., Chen D.W. (2016). Benzoic acid beneficially affects growth performance of weaned pigs which was associated with changes in gut bacterial populations, morphology indices and growth factor gene expression. J. Anim. Physiol. Anim. Nutr..

[44] Gossling J., Moore W.E.C. (1975). Gemmiger formicilis, n.gen., n.sp., an Anaerobic Budding Bacterium from Intestines. Int. J. Syst. Bacteriol..

[45] Vital M., Karch A., Pieper D.H., Shade A. (2017). Colonic Butyrate-Producing Communities in Humans: An Overview Using Omics Data. mSystems.

[46] Le Roy T., Moens de Hase E., Van Hul M., Paquot A., Pelicaen R., Regnier M., Depommier C., Druart C., Everard A., Maiter D. (2022). Dysosmobacter welbionis is a newly isolated human commensal bacterium preventing diet-induced obesity and metabolic disorders in mice. Gut.

[47] Louis P., Flint H.J. (2009). Diversity, metabolism and microbial ecology of butyrate-producing bacteria from the human large intestine. FEMS Microbiol. Lett..

[48] Pasolli E., Asnicar F., Manara S., Zolfo M., Karcher N., Armanini F., Beghini F., Manghi P., Tett A., Ghensi P. (2019). Extensive Unexplored Human Microbiome Diversity Revealed by Over 150,000 Genomes from Metagenomes Spanning Age, Geography, and Lifestyle. Cell.

[49] Firrman J., Friedman E.S., Hecht A., Strange W.C., Narrowe A.B., Mahalak K., Wu G.D., Liu L., Fraser C.M. (2024). Preservation of conjugated primary bile acids by oxygenation of the small intestinal microbiota in vitro. mBio.

