# Supplementary material for: The Effect of Sodium Benzoate on the Gut Microbiome Across Age Groups
Source: Foods. 2025 Aug 24;14(17):2949. doi: 10.3390/foods14172949 (PMC12428287; doi:10.3390/foods14172949)
Supplement: Supplementary file 1 [file foods-14-02949-s001.zip › Figure S1.pdf]

Supplemental Figure S1

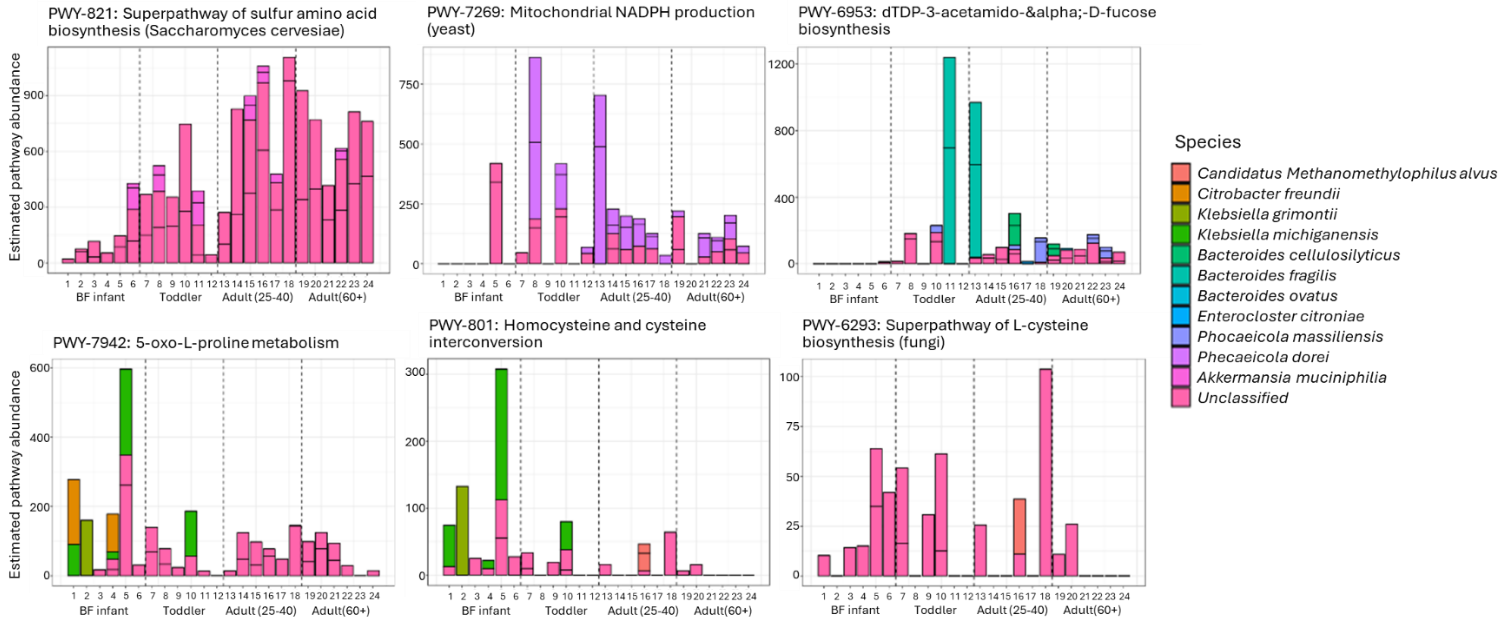

Figure S1: Species-level relative contribution of taxa that encode the 6 pathways significantly changed by SB that can be attributed to specific taxa, but not *E. coli*.
